# Status Epilepticus Caused by an Herbicide Poisoning

**DOI:** 10.1155/2019/3014138

**Published:** 2019-07-11

**Authors:** Mohammed Sidayne, Adnane Lahlou, Saïd Benlamkaddem, Mohamed Adnane Berdai, Mustapha Harandou

**Affiliations:** Obstetric and Pediatric Intensive Care Unit, Hassan II Academic Hospital, Fez, Morocco

## Abstract

Triclopyr is a pyridine derivative, widely used as an herbicide. It controls plant growth by interfering with plant growth hormones, auxins. It should have few effects in humans as these are nonexistent in mammals. It can prove however very severe in cases of acute poisoning.

## 1. Case Report

We report the case of a four-year-old child (weight: 15 kg, height: 100 cm) who was admitted to a local hospital for tonic-clonic seizures six hours after ingestion of an unknown quantity of an undetermined substance found in the father's work field. The seizures were terminated after diazepam administration before referral to our intensive care unit for further management.

On admission to our unit twelve hours following the ingestion, the patient was obnubilated with a Glasgow coma scale of 9(E2V2M5), pinpoint pupils, tachycardic at 170 beats per minute, polypneic: 38 breaths per minutes, and SpO2 96% on 15l oxygen. Fingertip glycemia was 0.95 g/l. Shortly after admission, the patient presented tonic-clonic seizures unresponsive to diazepam and phenobarbital, defining thus a refractory status epilepticus and warranting rapid sequence induction and ventilation. The infant was put on midazolam 0.3mg/kg/h with a low dose norepinephrine support to ensure a correct mean arterial pressure. Upon this afebrile status epilepticus, a complete workup was begun including head computed tomography (CT), transcranial Doppler (TCD), complete blood count, and comprehensive metabolic panel as well as a toxicological screening. Head CT showed mild cerebral edema, and TCD of the median cerebral artery found elevated pulsatility indexes: 1.4 on both sides and low end diastolic velocities 47.8 cm/s. Sedation was deepened and the child received 1 g/kg of mannitol and was put on valproic acid and clobazam.

An EEG was performed showing no status epilepticus.

Blood tests were unremarkable without any electrolyte abnormalities, and basic toxicological screening did not find any traces of alphachloralose or organophosphates.

**Figure 1:**
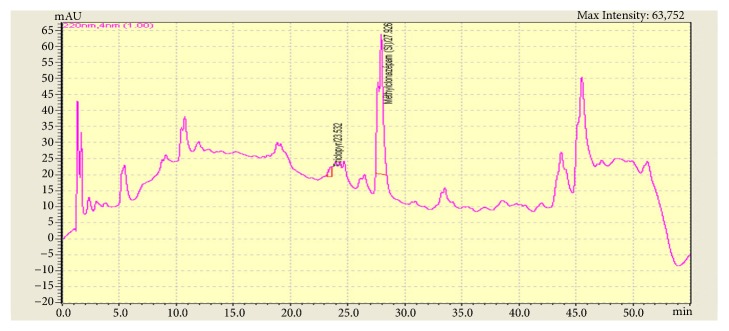
Chromatogram showing a triclopyr peak at 23rd minute, with methylclonazepam as internal standard.

Advanced toxicological analysis of the urine sample using high performance liquid chromatography-diode array detection identified a substance used in herbicides: triclopyr (Figure 1).

After two days of sedation and a normal transcranial Doppler, sedation was stopped with a complete neurologic recovery on day 3 of admission. Weaning from mechanical ventilation was however difficult due to ventilation associated pneumonia successfully treated with antibiotics. The patient was extubated on day seven and transferred to the pediatric ward for subsequent management.

## 2. Discussion

Herbicides are chemicals widely used in the agriculture industry for undesired plants extermination. They have numerous modes of action, among which are lipid biosynthesis inhibitors, photosynthesis inhibitors, and plant growth regulators [1]. Plant growth regulators, also known as synthetic auxins, belong to different chemical classes: phenoxy carboxylic acids, pyridine derivatives like triclopyr, benzoic acids, and carboxymethyl derivatives [2].

Triclopyr,(3,5,6-trichloro-2-pyridinyloxyacetic acid) is an organic compound widely used in the agriculture industry as an herbicide and a fungicide. It replaced in 1970 the then widely used 2,4,5-trichlorophenoxyacetic acid, another synthetic auxin banned due to toxicity issues [3, 4]. It is often formulated as a triethylamine salt (TEA) or butoxyethyl ester. Its toxicity has been studied in animals to establish the no* observed* effect level (NOEL) and the lowest observed effect level doses (LOEL) as well as the LD50. Pharmacokinetics were studied in human healthy volunteers, and it was found that the peak plasma level was reached after two hours and a half with a half-life of 5.1 hours [5]. Neurotoxicity has been studied in rodents and is thought to stem from decreased mRNA expression in neurons producing antioxidants [6]. Acute and chronic effects on humans have been reported as an occupational exposure; prolonged contact exposure may cause skin and eye irritation with moderate corneal injury [7]. Acute inhalation intoxication would not be expected due to a high LC50 [7]. Ingesting small amounts is unlikely to cause injuries; large amounts however might lead to gastrointestinal irritation [8].

Acute intoxication in humans is very rare whether accidental or suicidal. Only a handful of cases have been reported in the literature and ours is as far as we know the first case of such intoxication in children. There are, on the other hand, more studies regarding intoxication with chlorophenoxy acid herbicides which have the same mode of action as triclopyr albeit of another chemical class. Park et al. reported a case series of seventeen patients having ingested auxin-like herbicides, among which two male patients ingested triclopyr, with a death reported in a patient intoxicated with another herbicide (mecoprop) [9]. Guerin reported the case of a lethal ingestion in a patient with psychiatric history in whom the diagnosis was made postmortem [10]. In Kyong's case report, the evolution was favorable; the cardiac toxicity upon the intoxication was the main cause of concern [11]. Another case series regarding chlorophenoxy acid herbicides reported three cases of triclopyr ingestion with positive outcome after appropriate management of the ensuing renal failure and metabolic acidosis [12]. In a Sri Lankan case series involving 181 patients self-poisoned with 4-chloro-2-methylphenoxyacetic acid (MCPA), death rate was 4.4%. There was no correlation between MCPA levels and symptoms severity. Management included supportive measures and urine alkalinization [13]. Urine alkalinization is, per a 2007 Cochrane review, not supported by enough evidence to recommend its routine usage; it should be nevertheless taken into account as it may provide some benefit, especially in chlorophenoxy herbicides poisonings [14]. Regarding triclopyr, however, animal studies have shown that urinary pH has no effect on excretion due to its low pKa [15]. Hemodialysis has yet to be reported in triclopyr poisoning; it was however used alongside resin hemoperfusion in a Croatian case series involving four patients intoxicated with phenoxy carboxylic herbicides with positive outcome [16]. Pannu and associates report two cases of 2,4-dichlorophenoxyacetic acid treated successfully with intermittent hemodialysis [17].

In our country, organophosphate is the most common product involved in child poisoning with industrial products [18], a substance for which, unlike triclopyr and other herbicides, an antidote and management guidelines are available [19].

## 3. Conclusion

Intoxication with triclopyr is uncommon and should be managed adequately with intensive supportive measures as there is no antidote and no enough evidence to suggest a toxidrome and standardized management. We stress here again the fact that hazardous substances should be kept away from children to avoid such unfortunate events.
